# LINE-1 266/97 and ALU 260/111 Copy Number Ratios in Circulating Cell-Free DNA in Plasma as Potential Biomarkers for the Detection of Prostate Cancer: A Pilot Case-Control Study

**DOI:** 10.3390/ijms26188862

**Published:** 2025-09-11

**Authors:** Domenico Tierno, Nicola Pavan, Fabiola Giudici, Gabriele Grassi, Eleonora Valeri, Fabrizio Zanconati, Fabio Traunero, Giovanni Liguori, Bruna Scaggiante

**Affiliations:** 1 Department of Medicine, Surgery and Health Science, University of Trieste, 34129 Trieste, Italy; domenico.tierno@units.it (D.T.); ggrassi@units.it (G.G.); f.zanconati@fmc.units.it (F.Z.); 2 Department of Surgical, Oncological, and Oral Sciences, University of Palermo, 90127 Palermo, Italy; nicola.pavan@unipa.it; 3 Cancer Epidemiologic Unit, Centro di Riferimento Oncologico di Aviano (CRO), Istituto di Ricovero e Cura a Carattere Scientifico (IRCCS), 33081 Aviano, Italy; fabiola.giudici@cro.it; 4 Department of Life Sciences, University of Trieste, 34127 Trieste, Italy; s242241@ds.units.it; 5 Urological Clinic, Department of Medicine, Surgery and Health Sciences, University of Trieste, 34149 Trieste, Italy; fabio.tra92@gmail.com (F.T.); gliguori@units.it (G.L.)

**Keywords:** ALU, cfDNA, cfDI, copy number, cfDNA integrity, ddPCR, LINE-1, liquid biopsy, prostate cancer

## Abstract

Prostate cancer (PCa) is the second most common cancer and the fourth leading cause of cancer death in men worldwide. PSA screening for PCa diagnosis is not disease-specific; the discovery of novel and efficient biomarkers is therefore recommended. The concentration and integrity of circulating cell-free DNA (ccfDNA) in the blood of PCa patients could represent innovative and more specific tools for the clinical management of PCa. Digital droplet PCR (ddPCR) was used to determine the copy number ratio of ALU 260/111 bp and LINE-1 266/97 bp in the plasma of a cohort of 40 PCa and 18 BPH patients in a blinded prospective study. The amount of ccfDNA in the plasma of PCa and BPH patients was calculated from the *EEF1A2* and *ESR1* gene copy numbers. The ALU 260/111 and LINE-1 266/97 copy number ratios were significantly lower in the plasma of PCa patients compared to benign prostatic hyperplasia (BPH) ones (*p*-value; ALU 260/111: 0.006; LINE-1 266/97: 0.037). The area under the curve (AUC) indicated a good accuracy of two ratios and their product (ALU 260/111 * LINE 266/97, A*L) in discriminating PCa patients from BPH ones (AUC; ALU 260/111: 0.72; LINE-1 266/97: 0.67; A*L: 0.76). The ccfDNA concentration measured by *EEF1A2* and *ESR1* targets was significantly higher in the plasma of PCa patients compared to BPH patients, (*p*-value: *EEF1A2*, 0.017; *ESR1*, 0.024). The pilot ddPCR analysis of the ALU 260/111 and LINE-1 266/97 ratios in plasma indicates a new, reproducible and specific method for improving the early diagnosis of PCa. Further studies on larger cohorts are needed to confirm the results and clinical application.

## 1. Introduction

Prostate cancer (PCa) is one of the most common and deadly cancers in men worldwide [1]. According to the American Cancer Society, PCa will represent the second leading cause of cancer and cancer-related death in the United States, with an estimated 317,780 new cases and 35,770 new deaths in 2025 [2]. Currently, PSA screening is the most used blood test for detecting PCa. Although this technique significantly reduces PCa-specific mortality, the limitations of the PSA test in screening the population are well recognised and alternative specific and more informative tests are currently being investigated. Over the past three decades, PSA screening methods for PCa have proven to be problematic and can lead to overtreatment. In addition, the PSA test is not disease specific and may be elevated in patients with prostatitis and benign prostatic hyperplasia (BPH) [3]. On the other hand, a standard biopsy to diagnose prostate cancer is an unpleasant and sometimes painful procedure, with a detection rate of only about 50% [4].

The development of novel and more specific molecular biomarkers will be key to the early identification of PCa patients, and the monitoring of response, treatment resistance and relapse. In this context, minimally invasive blood-based “liquid” biopsies are attractive as a practical substitute for solid tissue, as they provide information on the diagnosis, prognosis and therapeutic response of the tumour. Components of liquid biopsies, such as circulating tumour cells and circulating cell-free tumour DNA, have shown remarkable potential to provide insights into PCa patient outcomes by detecting specific genomic and transcriptomic alterations [5]. In cancer, the analysis of circulating cell-free DNA includes the detection of circulating cell-free DNA (ccfDNA) quantity, the determination of the circulating cell-free DNA integrity (cfDI), the methylation rate of ccfDNA, the mutations and/or copy number aberrations of specific genes in circulating tumour DNA (ctDNA) [6]. The cfDI refers specifically to the assessment of ccfDNA fragmentation using genes or genomic repetitive elements as targets; for this purpose, it is calculated as the ratio between long and short targets within a selected sequence. The basic idea is that this ratio changes depending on the rate of cancer cell death and also on the type of cancer cell death, which may differ from that of normal cells [7]. CfDI has been shown to vary with different targets in breast cancer, and its potential diagnostic, prognostic and therapeutic value has been demonstrated in many papers utilising ALU or LINE repetitive sequences [8]. On the contrary, there is little information on the usefulness of the cfDI for repetitive elements in prostate cancer, and of these, only the cfDI of ALU has been studied [9]. The advantages of analysing ALU and LINE-1 cfDI are: (1) these biomarkers are not dependent on tumour mutations which limit the detection of cancer to selected populations that may or may not be present in tumour tissue; (2) they are highly abundant in the genome, which increases sensitivity; and (3) ALU and LINE-1 have been shown to play a role in cancer development and progression in prostate cancer [10,11,12,13,14]. Recently, our research group has shown that cfDI from ALU and LINE-1 can discriminate patients with early-stage primary breast cancer using a droplet digital PCR (ddPCR) assay [15]. In this pilot study, we aim to evaluate the settings of the highly sensitive and quantitative diagnostic platform ddPCR for the detection of ALU and LINE-1 copy number ratio and cfDI in plasma of a cohort of subjects who underwent biopsy for PCa surveillance. We also assessed the amount of ccfDNA in plasma of PCa patients compared to patients with BPH by the copy number of the *EEF1A2* gene, which is considered a target with reasonable accuracy for haploid genomes already analysed by ddPCR, as reported in our previous study on breast cancer [15]. As a further control, we calculated the amount of ccfDNA based on the copy number of the estrogen receptor 1 gene (*ESR1*). As a secondary endpoint, we investigated the correlation of the ALU and LINE-1 copy number ratio with the clinical-pathological status of the patients at baseline and twelve months after diagnosis.

## 2. Results

### 2.1. Characteristics of the Study Population

Patients admitted to the Urology Unit of Cattinara Hospital (Trieste) for a transrectal control biopsy of the prostate, were invited to participate in this prospective, blinded study. From November 2021 to May 2022, a total of 58 patients were enrolled in the study. The blood samples were taken before the biopsy. After analysis of the collected plasma, patients were divided into two groups based on their histological diagnosis: Patients with Benign Prostatic Hyperplasia (BPH) and patients with Prostate Cancer (PCa). We did not include a group with normal prostates as our cohort consisted of men referred for biopsy due to clinical suspicion and/or elevated PSA. In this setting, histological BPH is highly prevalent with increasing age (approximately 50% aged 51–60 years and ≥80–90% beyond 70), so truly normal tissue is rarely found [16,17].

The clinical characteristics of these patients are listed in Table 1.

The two groups were not homogeneous in terms of age, with the median (IQR) of age being 76.0 (66.8–79.3) and 68.0 (60.3–74.5) for the PCa group and the BPH group, respectively. This was not surprising as the American Cancer Society emphasises that the risk of developing PCa increases significantly with age, so it is more common in older men, particularly those over 50 [18]. To detect possible age-related biases, we analysed the correlation between age and each biomarker examined. The results are presented in each of the following subsections.

### 2.2. Evaluation of ALU 260/111 and LINE-1 266/97 Copy Number Ratio and cfDI in the Plasma of Patients’ Cohort

Fragmentation of ccfDNA was assessed in the plasma of the *patient cohort* prior to final diagnosis in a blinded manner using ddPCR by analysing the ratio between long and short fragments of ALU 266 bp over 111 bp (ALU 260/111) or of LINE-1 266 bp over 97 bp (LINE-1 266/97). The different ratio of short and long fragments of a given target has indeed been observed in many cancers, such as prostate cancer and breast cancer [19,20,21,22,23,24]. The population of the study was divided into PCa and BPH patients after the completion of the diagnosis. The analysis results were given as copy number ratio and as cfDI (ratio of fragment quantities), the latter being another ratio calculation described in the literature [22,23,25]. In addition, a Receiver Operating Characteristic (ROC) analysis assessed the diagnostic predictivity of ALU 260/111 and LINE-1 266/97 copy number ratios.

**Figure 1:**
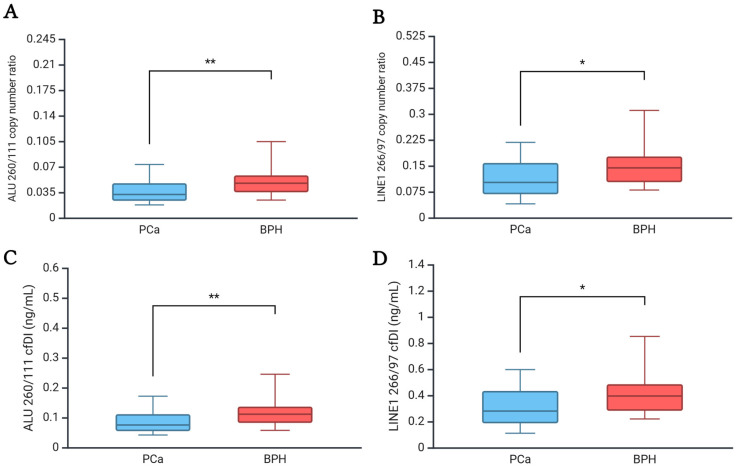
Box plots of ALU 260/111 copy number ratio (**A**), LINE-1 copy number ratio (**B**), ALU 260/111 cfDI (ng/mL) (**C**), and LINE-1 266/97 cfDI (ng/mL) (**D**) in plasma of PCa and BPH. * *p*-value < 0.05, ** *p*-value < 0.01. PCa patients n: 40; BPH patients n: 18. Abbreviations: BPH, Benign Prostatic Hyperplasia group; PCa, Prostate cancer group; n, number of patients. Created in BioRender. Scaggiante, B. (2025) https://BioRender.com/p9z892w (accessed on 22 July 2025).

**Figure 2:**
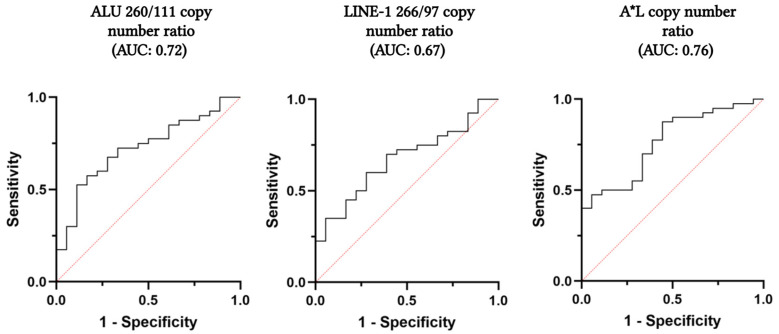
ROC curves and AUC of ALU 260/111 copy number ratio, LINE-1 266/97 copy number ratio, and their product (ALU260/111*LINE-1266/97). Abbreviations: A*L, ALU 260/111*LINE-1 266/97. Created in BioRender. Scaggiante, B. (2025) https://BioRender.com/vtmha4x (accessed on 22 July 2025).

As shown in Table 2 and Figure 1, a significant decrease in ALU 260/111 and LINE-1 266/97 copy number ratios was observed in the PCa group compared to the BPH group (ALU 260/111 PCa vs. BPH: median 0.03 vs. 0.05, *p*-value: 0.006; LINE-1 266/97 PCa vs. BPH: median 0.10 vs. 0.14, *p*-value: 0.037). The observed decrease was also significant in terms of cfDI (ALU 260/111 PCa vs. BPH: median 0.07 vs. 0.11, *p*-value = 0.007; LINE-1 266/97 PCa vs. BPH: median 0.28 vs. 0.39, *p*-value = 0.037). The ROC curves showed that both ALU 260/111 and LINE-1 266/97 copy number ratios have good accuracy in distinguishing PCa and BPH patients (AUC + 95% CI, ALU 260/111: 0.72, 0.59–0.86; LINE-1 266/97: 0.67, 0.53–0.81) (Figure 2). The optimal cut-off points for maximising the diagnostic predictive power of ALU 260/111 and LINE-1 266/97 were 0.0383 and 0.1326, respectively (ALU 260/111, Sensitivity: 67.5%, Specificity: 72.2%; LINE-1 266/97, Sensitivity: 70.0%; Specificity: 61.1%) (Table 3).

Moreover, we investigated the possible improvement of the diagnostic efficiency of the ALU 260/111 and LINE-1 266/97 copy number ratios by combining them in a composite score, as previously reported [22,26,27]. We found that the A*L score, obtained by multiplying the two copy number ratio values for each subject analysed (ALU 260/111 * LINE-1 266/97), improved diagnostic accuracy in distinguishing PCa from BPH patients. The resulting ROC curve showed a higher AUC of A*L compared to the individual ratios alone (AUC, A*L: 0.76, 0.64–0.89; ALU 260/111: 0.72, 0.59–0.86; LINE-1 266/97: 0.67, 0.53–0.81) (Figure 2). At the optimal cut-off point of 0.0069, the A*L composite score showed better sensitivity compared to the individual ALU 260/111 and LINE-1 266/97 ratios, and reduced the number of false negatives (FN) (FN, ALU 260/111: 13; LINE-1 266/97: 12; A*L: 5) (Table 3).

It is noteworthy that the ratios of ALU 260/111 and LINE-1 266/97 and their product A*L, do not correlate with age in either PCa or the BPH group (see Supplementary Materials, Table S1).

### 2.3. Evaluation of ccfDNA Quantity by EEF1A2 and ESR1 Copy Number in the Plasma of PCa and BPH Patients

**Figure 3:**
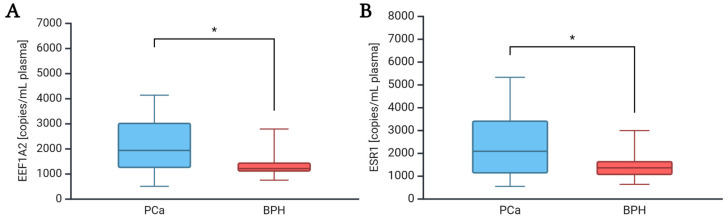
Box plots of EEF1A2 copy number (**A**) and ESR1 copy number (**B**) in plasma of PCa and BPH patients. * *p*-value < 0.05. PCa patients n: 40; BPH patients n: 18. Abbreviations: BPH, Benign Prostatic Hyperplasia group; PCa, Prostate cancer group; n, number of patients. Created in BioRender. Scaggiante, B. (2025) https://BioRender.com/7dj8r23 (accessed on 22 July 2025).

The amount of ccfDNA in the plasma of PCa and BPH groups was calculated by ddPCR based on the copy number of *EEF1A2* and *ESR1* genes, assuming that one copy of the target gene corresponds to one human haploid genome (3.3 pg) (see Materials and Methods). As shown in Table 4 and Figure 3, the copies per ml of plasma of both *EEF1A2* and *ESR1* were significantly higher in the PCa group compared to BPH (*EEF1A2* copies/mL plasma PCa vs. BPH: median 1925.0 vs. 1202.5, *p*-value: 0.017; *ESR1* copies/mL plasma PCa vs. BPH: median 2075.0 vs. 1351.0, *p*-value: 0.024). The ccfDNA quantity, calculated from both *EEF1A2* and *ESR1,* was also significantly higher in the PCa group compared to BPH (ccfDNA ng/mL from *EEF1A2* PCa vs. BPH: median 6.35 vs. 3.97, *p*-value: 0.017; ccfDNA ng/mL from *ESR1* PCa vs. BPH: median 6.85 vs. 4.45, *p*-value: 0.024) (Table 4). Of note, the ratio between the *EEF1A2* and *ESR1* copy number did not change significantly between the PCa and BPH groups (*EEF1A2/ESR1* PCa vs. BPH: median 0.91 vs. 0.92, *p*-value: 0.737) (Table 4). No significant correlations were observed between ccfDNA quantity, *EEF1A2* and *ESR1* copy number, and age, suggesting that this parameter had no effect on the difference between PCa and BPH (see Supplementary Materials, Table S2).

### 2.4. Correlation of ALU 260/111 and LINE-1 266/97 Copy Number Ratio with the Clinical-Pathological Status of PCa Patients

As a secondary endpoint of this study, we investigated the correlations between the clinical-pathological parameters of the PCa patients and the ALU 260/111 or LINE-1 266/97 copy number ratios at the start of the study and after one-year. Table 5 shows that no significant correlations were observed between the analysed parameters.

The correlation of our parameters with PSA levels at baseline or at one-year follow-up in the PCa and BPH groups is shown in Table 6. Only ALU 260/111 showed a positive correlation with PSA levels in PCa patients at baseline (Spearman coefficient: 0.313), although the correlation was not statistically significant (*p*-value: 0.06).

## 3. Discussion

Prostate cancer is the second leading cause of cancer and the sixth leading cause of cancer death in men worldwide [1]. In PCa, there is still a need to find biomarkers that can differentiate between malignant and benign diseases or health conditions in a non-invasive manner. Liquid biopsy is a minimally invasive technique of great clinical interest, particularly for tumour management. It is based on the analysis of tumour-derived elements, including cell-free DNA (ccfDNA), circulating tumour cells (CTCs), tumour-educated platelets (TEPs), and extracellular vesicles (EVs), in body fluids. Over the years, it has significantly reshaped the processes of tumour diagnosis, prognosis, and therapeutic decision-making [9].

In our study, we investigated the role of ccfDNA fragmentation by analysing the copy number ratio of long (260 bp and 266 bp) and short (111 bp and 97 bp) ALU and LINE-1 fragments in the plasma of a cohort of patients who had undergone transrectal prostate biopsy. After histological analysis, the patients were divided into two groups: those with Benign Prostatic Hyperplasia (BPH) and those with Prostate Cancer (PCa). The study was performed the using digital droplet PCR, a PCR platform in which DNA templates are split into thousands of nano-sized droplets, which improves the sensitivity and reproducibility of the amplification process.

ALU and LINE-1, together with HERV and SVA, represent the four primary classes of human retrotransposons, mobile DNA elements that make up about 45% of the human genome. LINE-1 and HERV encode their own reverse transcriptase, whereas ALU and SVA require the retrotranscription machinery of LINE-1 [28]. LINE-1 elements have a size of about 6000 base pairs in size and make up about 17% of the human genome mass [29]. In 50% of human cancers, there is a notable upsurge in LINE-1’s capacity for transposition, and this heightened activity has been linked to the insertion of LINE-1 elements into tumour suppressor genes and changes in their methylation patterns, which have implications for the development of malignancies [12]. ALUs are repetitive sequences, that are about 300 bp long, and make up about 11% of the total human genome mass [30]. Like LINE-1, the insertion of ALU sequences into gene promoters or gene coding regions, can lead to changes in gene expression and methylation patterns, that ultimately trigger tumour onset and progression [31].

To our knowledge, this is the first study to investigate the role of ALU 260/111 and LINE-1 266/97 ratios for PCa by liquid biopsy. Many studies have evaluated the plasma ALU 260/111 and LINE-1 266/97 ratios as diagnostic, prognostic, and predictive biomarkers for several tumours including breast cancer (BC). In BC, plasma ALU 260/111 and LINE-1 266/97 ratios were found to be lower compared to healthy controls [15,19]. Furthermore, low ratios were associated with worse progression-free survival (PFS) and overall survival (OS) [19], as well as a higher risk of tumour recurrence [32]. Another study has shown that these ratios were significantly increased in the plasma of patients undergoing neoadjuvant chemotherapy (NACT) compared to the corresponding plasma before NACT [33]. In this pilot study, the analysis of ALU 260/111 and LINE-1 266/97 copy number ratios, and ccfDNA quantification, was based on a robust ddPCR method, that ensures absolute quantification and reproducibility by generating approximately 20,000 independent replicates in the form of oil-generated droplets in each test sample. In addition, this system can be used to analyse fluorescent products in one and two dimensions, which makes it possible to distinguish between specific and non-specific amplification reactions. We found that copy number ratios of ALU 260/111 and LINE-1 266/97 and cfDI in plasma were significantly lower in PCa compared to BPH patients. Moreover, the ROC curves showed that the copy number ratios accurately discriminate between PCa and BPH patients (AUC ALU 260/111 = 0.72; AUC LINE-1 266/97 = 0.67). Interestingly, the composite score as a product of ALU 260/111 and LINE 266/97 (ALU 260/111 * LINE-1 266/97, A*L) increased the diagnostic accuracy of the ratios alone (AUC A*L = 0.76). These results are consistent with our previous study on BC [15] and with other similar studies [19,34,35]. The decrease in copy number ratios ALU 260/111 and LINE-1 266/97 indicates a higher fragmentation of ccfDNA in PCa than in BPH patients. The reasons for this can be multiple, including an increased apoptosis rate, genetic or epigenetic aberrations, and different nuclease expressions in cancer cells. All in all, these processes can change the ratio of DNA fragment sizes released in the bloodstream of cancer patients compared to individuals without cancer [36,37,38]. Of note, the absolute values of ALU 260/111 and LINE-1 266/97 copy number ratios achieved in this study on PCa were lower than those from our previous study on BC for both the PCa and the BPH group than the values from our previous study on BC (see Supplementary Materials, Table S3). This may be due to the individual’s gender or, more probably, to the different ccfDNA extraction methods (Magcore for BC and Maxwell for PCa). Indeed, several studies have reported that the extraction methods can influence the amount and size of ccfDNA isolated from a liquid biopsy [39,40]. Nevertheless, a significant decrease in plasma ALU 260/111 and LINE-1 266/97 copy number ratios was observed in cancer patients compared to controls in both our studies on PCa and BC (see Supplementary Materials, Table S3).

One of the major limitations of our current research is the limited number of participants in the pilot study. Moreover, our population lacks a consistent number of patients with advanced and metastatic PCa tumour stages, with a focus on early-stage PCa (number of patients: Stage I–II = 30, Stage III–IV = 8). Further studies in a larger and more heterogeneous cohort of PCa patients will be necessary to validate our results. The extension of the study to the healthy population is another important point in order to deepen the diagnostic values of the biomarkers. However, the fact that we found no age-dependence for the biomarkers analysed and used a highly reproducible method, supports this finding and future studies in a larger cohort to confirm the ALU 260/111 and LINE-1 266/97 copy number ratios as biomarkers for prostate cancer detection. It is important to point out that in the literature, ccfDNA fragmentation studies using ALU and LINE-1 sequences have been performed by targeting long and short fragments of different sizes, which in turn affects the outcome of the analysis. For example, the LINE-1 259/97 ratio in the plasma of BC patients was found to be higher than in patients with benign breast disease (BBD) and healthy controls. This ratio also decreased in the plasma of patients’ after adjuvant chemotherapy (ACT) compared to before ACT [41]. Other studies have instead shown that the ALU 247/115 ratio in the plasma of PCa and BC patients was higher than in patients with benign disease and healthy controls [20,21,22,23,24,42]. While these results confirm the efficacy of ALU and LINE-1 cfDI as tumour biomarkers, they differ from those we and others have obtained with ALU 260/111 and LINE-1 266/97 ratios. We believe that qPCR represents a gold standard for the amplification of both long and short fragments [9], but the choice of a fragment of a size can underestimate or overestimate the concentration of short or long DNA fragments in the liquid biopsy. It is therefore necessary to facilitate the standardisation of ALU and LINE-1 plasma analysis before it can be meaningfully used in routine clinical practice. The copy numbers of *EEF1A2* and *ESR1*, and the ccfDNA concentration calculated from them, were higher in the plasma of PCa patients compared to BPH ones. These results are consistent with many studies indicating a higher concentration of ccfDNA in cancer patients compared to patient with benign diseases or healthy individuals; this is likely due to an increased rate of apoptotic and necrotic cell death in cancer cells [41,43,44,45,46]. Moreover, the *EEF1A2/ESR1* ratio showed no significant differences between the PCa and BPH groups, suggesting that, on average, there were no gene copy number variations between the PCa and BPH groups in these target genes, ensuring the accuracy of the ccfDNA quantity calculation. Also worth mentioning is the correlation analysis (Spearman test) performed between *EEF1A2*, *ESR1*, ALU 260/111, and LINE-1 266/97 in the plasma of PCa patients (see Supplementary Materials, Table S4). The ALU 260/111 copy number ratio showed a significant negative correlation with *EEF1A2* and *ESR1* copy number (ALU 260/111 vs. EEF1A2: −0.368, *p*-value = 0.02; ALU 260/111 vs. *ESR1*: −0.323, *p*-value = 0.04), which means that a decrease in the ALU 260/111 ratio was associated with an increase in *EEF1A2* and *ESR1* copy number (and thus with an increase in ccfDNA concentration) in the plasma of PCa patients. A negative correlation was also observed between the LINE-1 266/97 copy number ratio and the *EFF1A2* or *ESR1* copy numbers in the plasma of PCa patients, but statistical significance was only reached for *ESR1* (LINE-1 266/97 vs. *ESR1*: −0.373, *p*-value: 0.02). These results suggest that the ccfDNA concentration in the plasma of PCa patients may be associated with an increase in ccfDNA fragmentation.

Finally, our results showed no significant correlation between ALU 260/111 and LINE-1 266/97 copy number ratio and the clinical-pathological features of PCa patients neither at diagnosis nor at 1-year follow-up. This is probably due to the limited number of patients analysed in this pilot study. In addition, the lack of statistical significance for the parameters recurrence and tumour stage, could be related to the low heterogeneity in terms of the number of patients in the categorical groups studied. Indeed, there are only four PCa patients with tumour recurrence and eight PCa patients with advanced tumour stages (III and IV). However, these preliminary findings do not rule out the possibility that ALU and LINE-1 biomarkers may also be useful in distinguishing between aggressive and non-aggressive cancers and metastasis. In colorectal cancer cell lines, it was found that the accumulation of non-coding ALU RNA activates the epithelial–mesenchymal transition, and increases the metastatic potential of cancer cells [47]. Rigorous clinical trials and long-term follow-up studies will be crucial to determine the reliability of the biomarkers and their potential impact on patient outcomes within the realm of prostate cancer management. As we move into the uncharted territory of using ALU 266/97 and LINE-1 266/97 in liquid biopsy as a prognostic tool, further investigation is essential to validate the efficacy of the biomarkers and provide a solid foundation for its integration into clinical practice.

## 4. Materials and Methods

### 4.1. Study Population

The study was approved by the Ethics Committee of the University of Trieste (n. 110, 25 January 2021). From November 2021 to May 2022, we enrolled 58 patients who underwent prostate biopsy for suspected prostate cancer at the Urology Unit at the University of Trieste, Cattinara Hospital. Exclusion criteria were patients unable to sign a consent form and patients with active cancer under treatment.

At our centre, we routinely perform a blood test to assess blood clotting, haemoglobin levels, and blood cell count. During this period, we collected 12 mL of venous blood from every enrolled patient for liquid biopsy. The signature of consent and the blood sample collection were performed before the diagnosis of prostate cancer, so the enrolment in this study must be considered double-blinded. We enrolled 58 male patients eligible for the study with a mean age of 72 y.o. and a mean PSA level of 6.6 ng/mL before biopsy.

After histological diagnosis of prostate cancer and signing of informed consent, patients underwent standard staging procedures, including imaging and laboratory tests, according to international guidelines to define the extent of disease and support therapeutic decision-making https://uroweb.org/guidelines/prostate-cancer, accessed on 25 April 2025). Treatment strategies, including active surveillance, surgery, radiotherapy, and/or androgen deprivation therapy, were applied according to the individual clinical profile, tumour characteristics, and patient preferences, in accordance with EAU recommendations (https://uroweb.org/guidelines/prostate-cancer; https://doi.org/10.6004/jnccn.2024.0024, accessed on 25 April 2025). After completion of primary treatment, patients entered a structured follow-up programme according to EAU guidelines, which included serial PSA measurements and, if indicated, imaging studies to monitor for biochemical recurrence or progression of the disease (https://uroweb.org/guidelines/prostate-cancer, accessed on 25 April 2025; https://doi.org/10.1016/j.eururo.2013.09.002). The follow-up period for this study was terminated at the end of the enrolment phase to ensure consistency of observation across the cohort.

### 4.2. Plasma Preparation and ccfDNA Extraction

Blood was collected by venipuncture into a 10 mL Vacutainer K2-EDTA tube (Becton Dickinson, Franklin Lakes, NJ, USA) and processed within one hour from collection. The blood was centrifuged at 3000× *g* for 10 min at 4 °C, and the supernatant was then centrifuged at 12,000× *g* for 5 min at 4 °C. Plasma was aliquoted into 1 mL volumes in cryovials and stored at −80 °C.

Circulating cell-free nucleic acids (ccfNAs) were extracted from the plasma samples (1 mL) using the Maxwell^®^ RSC ccfDNA Plasma Kit (50) (Promega, Madison, WI, USA) according to the manufacturer’s instructions. The kit enables high-quality purification of ccfDNA using magnetic beads and without pretreatment steps. The purified ccfDNA was collected in 50 µL RNase-free H_2_O and stored at −80 °C for subsequent analysis.

### 4.3. ddPCR Analysis on EEF1A2, ESR1 and ccfDNA Quantity

The quantification of the copy number of *ESR1* and *EEF1A2* genes in the plasma of patients was performed using digital droplet PCR (ddPCR), a digital PCR method in which DNA templates are partitioned into thousands of nanolitre-sized droplets via a water-oil emulsion. An independent nano-reaction takes place within each droplet, allowing a massive number of parallel PCR amplifications of DNA templates. The high partitioning of the PCR reactions and the low sample requirement improve the sensitivity, specificity, and reproducibility of ddPCR compared to other PCR techniques.

The *EEF1A2* copy number in plasma was quantified by ddPCR Gene Expression Assay: *EEF1A2* (Biorad, Hercules, CA, USA). For each sample, the reaction mixture consisted of 10 µL ddPCR Supermix for Probes (No dUTP) (Biorad), 0.5 µL *EEF1A2* FAM assay (Biorad, contains the probe and primers), 5.5 µL RNAse-free water and 4 µL of the sample. The *ESR1* copy number in plasma was quantified using a mix of ddPCR Mutation Detection Assay (Biorad) for the following mutations of the *ESR1* gene: Y537C, Y537N, Y537S, and D538G. The kit was able to detect both wild-type (WT) and mutant (MT) sequences in the same sample using two targeting probes with different fluorochromes: FAM for WT and HEX for MT. Our group used this mix in a previous analysis to determine the mutational status of *ESR1* in the plasma of BC patients. The samples of this study were analysed as control. For each sample, the reaction mixture consisted of 10 μL ddPCR Supermix for Probes (No dUTP) (Biorad), 0.25 μL Y537C *ESR1* assay, 0.25 μL Y537N *ESR1* assay, 0.25 μL Y537S *ESR1* assay, 0.25 μL D538G *ESR1* assay (Biorad, each with probes and primers for both WT and MT sequences), 5 μL RNAse-free water and 4 μL of the sample. The *ESR1* mutations Y537C, Y537N, Y537S, and D538G were not found in any of the patients’ plasmas. Therefore, the FAM-positive *ESR1* WT droplets were considered as another target gene for our analysis of ccfDNA quantity. For both *EEF1A2* and *ESR1* quantification, partitioning of DNA templates was performed by the QX200™ Droplet Generator (Biorad) using 70 µL of Droplet Generation Oil for Probes solution (Biorad). The amplification conditions for EEF1A2 were 95 °C for 10 min, followed by 39 cycles of 94 °C for 30 s and 57 °C for 1 min (with a ramp rate of 2 °C/s), and then 98 °C for 10 min. The amplification conditions for ESR1 were 95 °C for 10 min, followed by 39 cycles of 94 °C for 30 s and 55 °C for 1 min (with a ramp rate of 2 °C/s), and then 98 °C for 10 min. Finally, droplet screening was performed using the QX200 Droplet Digital System (Biorad), which assigns a value of 0 (no fluorescence recorded, indicating absence of template) or 1 (fluorescence recorded, indicating presence of template) to each droplet. The software associated with the droplet reader system, Quantasoft (Biorad, vs. 1.7.7.0917), calculates the number of DNA template copies per µL of reaction volume by applying the Poisson algorithm to the number of positive and negative droplets. Specifically, this value is given by the formula:

λ = −log (1 − p)

where λ is the average number of copies for droplet and p is the ratio between the positive and negative droplets [48]. The copies per microliter of reaction volume are then obtained by knowing the average volume of each droplet (approximately 1 nL, as calculated by the software itself). Then, the number of copies in 20 µL (total reaction volume) was divided by 4 (sample volume used for ddPCR) and further divided by 20 (concentration factor of the Maxwell^®^ RSC ccfDNA Plasma Kit, Promega) to obtain the number of DNA template copies per µL of plasma. This value was then multiplied by 1000 to obtain the copy number for 1 mL of plasma.

The quantification of ccfDNA in plasma by *EEF1A2* and *ESR1* copy number was performed, assuming that one target gene copy corresponds to one haploid human genome, which is approximately 3.3 pg of DNA [49]. Accordingly, the ccfDNA quantity was calculated by the following formulae:

$$ccfDNA\;(EEF1A2)(\frac{ng}{mL})=\frac{ncEEF1A2\;*\;3.3}{1000}$$

$$ccfDNA\;(ESR1)(\frac{ng}{mL})=\frac{ncESR1\;*\;3.3}{1000}$$

where ncEEF1A2 and ncESR1 are the copy numbers of, respectively, *EEF1A2* and *ESR1* for 1 mL of plasma, 3.3 the weight of one haploid human genome in pg, and 1000 the conversion factor of pg in ng.

### 4.4. ddPCR Analysis on ALU 260, ALU 111, LINE-1 266 and LINE-1 97 Fragments

The copy numbers of ALU 260 bp, ALU 111 bp, LINE-1 266 bp, and LINE-1 97 bp in the plasma of PCa and BPH patients were determined by ddPCR using the EvaGreen assay (Bio-Rad) according to the manufacturer’s instructions. Due to the abundance of larger and shorter fragments, DNA samples were diluted as follows to optimise the ddPCR analysis: 1:60 for ALU 260, 1:540 for ALU 111, 1:20 for LINE-1 266 and 1:120 for LINE-1 97. The primer sequences were the same as those of Madhavan et al. [19]. For ALU fragments quantification, two different mixes were carried out: 10 µL QX200™ ddPCR™ EvaGreen Supermix (biorad), 1 µL Primer PCR Custom Assay ALU 260 (Biorad, 250 nM), 7 µL RNase-free water and 2 µL of the sample (for ALU 260); 10 µL QX200™ ddPCR™ EvaGreen Supermix (biorad), 0.5 µL Primer PCR Custom Assay ALU 111 (Biorad, 125 nM), 7.5 µL RNase-free water and 2 ul of the sample (for ALU 111). For LINE-1 fragments quantification, the two different mixes were: 10 µL QX200™ ddPCR™ EvaGreen Supermix (biorad), 1 µL Primer PCR Custom Assay LINE-1 266 (Biorad, 250 nM), 7 µL RNase-free water and 2 µL of the sample (for LINE-1 266); 10 µL QX200™ ddPCR™ EvaGreen Supermix (biorad), 0.5 µL Primer PCR Custom Assay LINE-1 97 (Biorad, 125 nM), 7.5 µL RNase-free water and 2 ul sample (for LINE-1 97). Partitioning of DNA templates was performed using the QX200™ Droplet Generator (Bio-Rad) with 70 μL QX200™ Droplet Generation Oil for EvaGreen solution (Bio-Rad). The amplification conditions were 95 °C for 5 min, followed by 40 cycles of 95 °C for 30 s and 56.5 °C for 1 min, and then 4 °C for 5 min and 90 °C for 5 min. The plate was then placed in the QX200 system for droplet reading, as described in the previous section.

A further ratio between the amount of the larger and shorter fragments of ALU and LINE-1, expressed in ng/mL plasma, was determined to compare the results with those reported in the literature. The following formulae were used to convert the copy numbers of ALU and LINE-1 fragments into DNA quantities:

ALU cfDI (ng/mL): (copies/mL of plasma ALU 260 × bpn × 618 × 1.7 × 10^−15^)/(copies/mL of plasma ALU 111 × bpn × 618 × 1.7 × 10^−15^)

LINE-1 cfDI (ng/mL): (copies/mL of plasma LINE-1 266 × bpn × 618 × 1.7 × 10^−15^)/(copies/mL of plasma LINE-1 97 × bpn × 618 × 1.7 × 10^−15^)

where cfDI is the ratio between LINE-1 266-LINE-1 97 and ALU 260-ALU 111 amount expressed in ng/mL plasma, bpn is the number of base pairs of the amplicon (266 bp for LINE-1 266, 97 bp for LINE-1 97, 260 bp for ALU 260, and 111 bp for ALU 111), 618 is the average weight of a base pair in daltons, and 1.7 × 10^−15^ is the conversion factor from daltons to ng DNA. Since the last two factors in the numerator and denominator are the same, the simplified formulae were as follows:

cfDI LINE-1 = (copies/mL of plasma LINE-1 266 × 266)/(copies/mL of plasma LINE-1 97 × 97)

cfDI ALU = (copies/mL of plasma ALU 260 × 260)/(copies/mL of plasma ALU 111 × 111)

### 4.5. Statistical Analysis

Categorical variables were presented as absolute values (percentages), and continuous variables as medians and interquartile ranges [IQR]. Normality was assessed using the Shapiro–Wilk test. Continuous variables were compared using the student’s or Mann–Whitney tests (and Kruskal–Wallis tests), depending on data distribution and the number of groups. Receiver operating characteristic (ROC) curves were generated to obtain the values of the area under the curve (AUC), with 95% CI, to determine which biomarker was the most reliable in identifying prostate cancer. For the prostate cancer patients, Spearman correlation coefficients were calculated to assess the relationship (i) between ALU 260/111 or LINE-1 266/97 copy number ratio and the other biomarkers and (ii) between age and the biomarkers. The significance threshold was set at *p* < 0.05.

The data were analysed using R statistical software (R version 4.2.3, R Foundation for Statistical Computing, Vienna, Austria. URL https://www.R-project.org/).

## 5. Conclusions

This study represents the first investigation of ALU 260/111 and LINE-1 266/97 in the plasma of PCa and BPH patients, and paves the way for groundbreaking insights into their applicability and reliability in a clinical context.

The ALU 260/111 and LINE-1 266/97 copy number ratio in plasma appear to be valuable biomarkers that could be developed for screening patients for prostate cancer. These non-invasive, highly reproducible ddPCR blood tests could help to better define the diagnosis and differentiate prostate cancer patients from benign lesions or healthy conditions. If the test is validated in a large cohort, it could enter into clinical practise to reduce the need for biopsy in patients, thereby reducing healthcare costs and patient suffering. In addition, the test could potentially be developed as a tool for molecular stratification of patients in follow-up care. It should be noted that this study has demonstrated the potential of ALU and LINE-1 targets as diagnostic biomarkers for Prostate cancer (PCa) in liquid biopsy, but the limited number of patients and the lack of heterogeneity in clinical subgroups hinder the adequate evaluation of their prognostic potential. In addition, the substantial amount of ALU and LINE-1 targets in ccfDNA, offers unique opportunities for the study of plasma biomarkers in liquid biopsies in cancer, including PCa. Overall, these results are encouraging and deserve further investigation with a larger cohort studied at diagnosis and follow-up to clarify the clinical significance of ALU 260/111 and LINE-1 266/97 copy number ratios in PCa.

## Figures and Tables

**Table 1:** Clinical characteristics of the study population. * *p*-value < 0.05. Abbreviations: BPH, Benign Prostatic Hyperplasia (BPH) IQR, interquartile range; n, number of patients; nd, no data; PCa, Prostate Cancer.

Variable	PCa Patients (n = 40)	BPH Patients (n = 18)	*p*-Value
Age median (IQR)	76.0 (66.8–79.3)	68.0 (60.3–74.5)	0.028 *
BMI mean (IQR)	25.6 (23.6–28.5)	25.6 (24.0–27.3)	0.942
PSA baseline median (IQR)	6.8 (4.6–9.5)	7.5 (5.5–11.6)	0.542
Tumour Stage number of patients (percentage)			
I–II (early)	30 (75.0%)	nd	nd
III–IV (advanced/metastatic)	8 (20.0%)	nd	nd
Not Available	2 (5.0%)	nd	nd

**Table 2:** Copy number ratio and cfDI of ALU 260/111 and LINE-1 266/97 in PCa and BPH patients. * *p*-value < 0.05, ** *p*-value < 0.01. Abbreviations: BPH, Benign Prostatic Hyperplasia; IQR, interquartile range; n, number of patients; PCa, prostate cancer.

Variable	PCa Patients (n = 40)	BPH Patients (n = 18)	*p*-Value
ALU 260/111 copy number ratio median (IQR)	0.03 (0.02–0.05)	0.05 (0.04–0.05)	0.006 **
LINE-1 266/97 copy number ratio median (IQR)	0.10 (0.07–0.14)	0.14 (0.10–0.17)	0.037 *
ALU 260/111 cfDI (ng/mL) median (IQR)	0.07 (0.06–0.11)	0.11 (0.08–0.13)	0.006 **
LINE-1 266/97 cfDI (ng/mL) median (IQR)	0.28 (0.19–0.43)	0.39 (0.29–0.48)	0.037 *

**Table 3:** Optimal cut-off point to improve the diagnostic predictivity of ALU 260/111 and LINE-1 266/97 copy number ratios and their product (ALU260/111*LINE-1266/97). Abbreviations: 95% CI, 95% confidence interval; A*L, ALU260/111*LINE-1266/97; FN, false negative; FP, false positive; PPV, positive predictive value; NPV, negative predictive value.

	ALU 260/111 Copy Number Ratio	LINE 266/97 Copy Number Ratio	A*L Copy Number Ratio
Cut-off	0.0383	0.1326	0.0069
Sensitivity (95% CI)	67.5% (50.9–81.4%)	70.0% (53.5–83.4%)	87.5% (73.2–95.8%)
Specificity (95% CI)	72.2% (46.5–90.3%)	61.1% (35.7–82.7%)	55.6% (30.8–78.5%)
PPV (95% CI)	84.4% (64.4–91.9%)	80.0% (58.6–89.6%)	81.4% (60.9–93.5%)
NPV (95% CI)	50.0% (33.3–78.2%)	47.8% (31.1–73.6%)	66.7% (43.8–85.4%)
FP	5	7	8
FN	13	12	5

**Table 4:** Quantification of *EEF1A2*, *ESR1*, *EEF1A2/ESR1* ratio and ccfDNA in plasma of PCa and BPH patients. * *p*-value < 0.05. Abbreviation: BPH, Benign Prostatic Hyperplasia; IQR, interquartile region; n, number of patients; PCa, prostate cancer.

Variable	PCa Patients (n = 40)	BPH Patients (n = 18)	*p*-Value
*EEF1A2* copies/mL plasma median (IQR)	1925.0 (1250.0–3000.0)	1202.5 (1106.3–1418.8)	0.017 *
*ESR1* copies/mL plasma median (IQR)	2075.0 (1131.3–3393.8)	1351.0 (1062.5–1618.8)	0.024 *
*EEF1A2/ESR1* median (IQR)	0.91 (0.76–1.07)	0.92 (0.86–1.03)	0.737
ccfDNA (ng/mL) by *EEF1A2* copy number median (IQR)	6.35 (4.12–9.9)	3.97 (3.65–4.68)	0.017 *
ccfDNA (ng/mL) by *ESR1* copy number median (IQR)	6.85 (3.73–11.20)	4.45 (3.51–5.34)	0.024 *

**Table 5:** Analysis of ALU 260/111 and LINE-1 266/97 copy number ratios according to age, BMI, tumour stage, ISUP group grade and recurrence in the group of patients. The data are reported as median. In parenthesis the interquartile range. * n = 2 not available data; ** n = 3 not available data. Abbreviations: n, number of patients.

Variable	ALU 260/111 Copy Number Ratio	*p*-Value	LINE-1 266/97 Copy Number Ratio	*p*-Value
Age				
<76 (n = 19)	0.03 (0.02–0.04)	0.212	0.10 (0.08–0.18)	0.316
≥76 (n = 21)	0.03 (0.02–0.05)		0.10 (0.06–0.13)	
BMI				
<25 (n = 15)	0.03 (0.02–0.04)	0.355	0.10 (0.08–0.15)	0.774
≥25 (n = 23)	0.03 (0.03–0.05)		0.10 (0.07–0.16)	
Tumour Stage *				
I-II (n = 30)	0.03 (0.02–0.05)	0.322	0.11 (0.08–0.17)	0.235
III-IV (n = 8)	0.04 (0.03–0.04)		0.09 (0.03–0.14)	
GLEASON score (ISUP) *				
Low risk (0–1) (n = 10)	0.03 (0.02–0.05)	0.159	0.10 (0.06–0.13)	0.515
Intermediate risk (2–3) (n = 18)	0.03 (0.02–0.03)		0.11 (0.08–0.18)	
High risk (4–5) (n = 10)	0.04 (0.03–0.05)		0.10 (0.08–0.13)	
Recurrence **				
Yes (n = 4)	0.04 (0.03–0.06)	0.325	0.11 (0.08–0.17)	0.575
No (n = 31)	0.03 (0.02–0.04)		0.11 (0.10–0.12)	

**Table 6:** Correlation analysis between ALU 260/111 and LINE-1 266/97 copy number ratio, and the PSA levels at baseline of PCa and BPH group. In parenthesis are reported the *p*-value. Abbreviations: BPH, Benign Prostatic Hyperplasia; PCa, Prostate cancer; nd, no data.

Variable vs. PSA Levels	PCa Patients (n = 38)	BPH Patients (n = 17)
ALU 260/111 copy number ratio baseline (*p*-value)	0.313 (0.06)	0.167 (0.523)
LINE-1 266/97 copy number ratio baseline (*p*-value)	−0.04 (0.802)	−0.06 (0.823)
ALU 260/111 copy number ratio one-year follow-up (*p*-value)	−0.004 (0.983)	nd
LINE-1 266/97 copy number ratio one-year follow-up (*p*-value)	−0.168 (0.332)	nd

## Data Availability

The data presented in this study are available on request from the corresponding author. Not viable for privacy.

## Supplementary Materials

The following supporting information can be downloaded at: https://www.mdpi.com/article/10.3390/ijms26188862/s1.
