# Supplementary material for: LINE-1 266/97 and ALU 260/111 Copy Number Ratios in Circulating Cell-Free DNA in Plasma as Potential Biomarkers for the Detection of Prostate Cancer: A Pilot Case-Control Study
Source: Int J Mol Sci. 2025 Sep 11;26(18):8862. doi: 10.3390/ijms26188862 (PMC12470076; doi:10.3390/ijms26188862)
Supplement: Supplementary file 1 [file ijms-26-08862-s001.zip › ijms-3806038-supplementary.pdf]

Table S1: Correlation analysis between ALU 260/111, LINE-1 266/97 copy number ratios or their product (ALU260/111*LINE-1266/97), and the individuals' age. In the table, the correlation coefficients are reported, while the p-values are reported in parentheses. Abbreviations: BPH, Benign Prostatic Hyperplasia; A*L, ALU260/111*LINE-1266/97; n, number of patients; PCa, Prostate Cancer.

Variable vs. Age	PCa Patients (n = 40)	BPH Patients (n = 18)
ALU 260/111 copy number ratio (*p*-value)	0.125 (0.443)	0.02 (0.929)
LINE-1 266/97 copy number ratio (*p*-value)	-0.162 (0.520)	-0.134 (0.409)
A*L copy number ratio (*p*-value)	-0.106 (0.516)	-0.04 (0.834)

Table S2: Correlation analysis between *EEF1A2*, *ESR1* or *EEF1A2*/*ESR1* copy number, and age. In the table, the correlation coefficients are reported, while the p-values are reported in parentheses. Abbreviations: BPH, Benign Prostatic Hyperplasia; PCa, Prostate Cancer

Variable vs. Age	PCa Patients (n = 40)	BPH Patients (n = 18)
EEF1A2 copies/mL plasma (*p*-value)	0.168 (0.300)	0.001 (0.999)
ESR1 copies/mL plasma (*p*-value)	0.167 (0.301)	-0.05 (0.851)
EEF1A2/*ESR1* copy number (*p*-value)	0.018 (0.910)	-0.105 (0.677)

Table S3: Comparison of ALU 260/111 and LINE-1 266/97 copy number medians between plasma of breast cancer patients and corresponding Healthy Controls (HC) from our previous study [1] and plasma of prostate cancer patients (PCa) and corresponding Benign Prostatic Hyperplasia (BPH) controls of our current study. Also, the copy number ratio between BC and PCa and HC and BPH are reported.

			**Ratio**
ALU 260/111	BC 0.079	PCa 0.03	BC/PCa 2.63
ALU 260/111	HC 0.093	BPH 0.05	HC/BPH 1.86
LINE-1 266/97	BC 0.19	PCa 0.10	BC/PCa 1.90
LINE 266/97	HC 0.27	BPH 0.14	HC/BPH 1.93

Table S4: Correlation matrix between ALU 260/111 copy number ratio, LINE-1 266/97 copy number ratio, *EEF1A2* copy number and *ESR1* copy number in plasma of PCa patients. * *p*-value <0.05, *** *p*-value < 0.001

	**ALU 260/111**	**LINE-1 266/97**	***EEF1A2***	***ESR1***
**ALU 260/111 copy number ratio (*p*-value)**	1.0	0.184 (0.254)	-0.323 (0.04)*	-0.368 (0.02)*
**LINE-1 266/97 copy number ratio (*p*-value)**	0.184 (0.254)	1.0	-0.373 (0.02)*	-0.172 (0.289)
**EEF1A2 copies/mL plasma (*p*-value)**	-0.368 (0.02)*	-0.172 (0.289)	1.0	0.709 (0.0001)***
**ESR1 copies/mL plasma (*p*-value)**	-0.323 (0.04)*	-0.373 (0.02)*	0.709 (0.0001)***	1.0

References

[1] Bortul, M.; Giudici, F.; Tierno, D.; Generali, D.; Scomersi, S.; Grassi, G.; Bottin, C.; Cappelletti, M.R.; Zanconati, F.; Scaggiante, B. A Case–Control Study by DdPCR of ALU 260/111 and LINE-1 266/97 Copy Number Ratio in Circulating Cell-Free DNA in Plasma Revealed LINE-1 266/97 as a Potential Biomarker for Early Breast Cancer Detection. *Int J Mol Sci* **2023**, *24*, doi:10.3390/ijms24108520.
